# Genetic Biomarkers and Circulating White Blood Cells in Osteoarthritis: A Bioinformatics and Mendelian Randomization Analysis

**DOI:** 10.3390/biomedicines13010090

**Published:** 2025-01-02

**Authors:** Yimin Pan, Xiaoshun Sun, Jun Tan, Chao Deng, Changwu Wu, Georg Osterhoff, Nikolas Schopow

**Affiliations:** 1 Department of Neurosurgery, Xiangya Hospital, Central South University, Changsha 410017, China; 2 National Clinical Research Center for Geriatric Disorders, Xiangya Hospital, Central South University, Changsha 410017, China; 3 Department of Plastic and Cosmetic Surgery, Xiangya Hospital, Central South University, Changsha 410017, China; 4 Department of Orthopedics, Trauma and Plastic Surgery, University Hospital Leipzig, 04103 Leipzig, Germany

**Keywords:** osteoarthritis, circulating white blood cell, neutrophils, biomarker, mendelian randomization

## Abstract

**Background**: Osteoarthritis (OA) is a prevalent degenerative joint disease that causes disability and diminishes quality of life. The pathogenesis of OA remains poorly understood, creating an urgent need for biomarkers to aid research, diagnosis, and treatment. **Methods**: This study integrated transcriptome data from the GEO database with bioinformatics analyses to identify biomarkers associated with OA. The bioinformatics methods utilized include the Limma package, WGCNA, PPI network analysis, and machine learning algorithms. Genetic variants were used as instrumental variables to evaluate the potential causal impact of circulating white blood cell (WBC) counts on OA. Data sources encompassed the largest genome-wide analysis for OA and a comprehensive GWAS summary for circulating WBC counts. Four mendelian randomization (MR) methods were employed to investigate the genetic association, with a primary focus on findings from the inverse variance-weighted (IVW) method. **Results**: Total of 233 OA-related genes were identified, showing significant enrichment in pathways associated with WBC function. Key biomarkers, including *CD4*, *CSF1R*, and *TYROBP*, were upregulated in OA samples and exhibited strong diagnostic potential. MR analysis findings provided evidence of a genetic association between elevated neutrophil counts and a reduced risk of OA across sites (IVW: OR = 0.97, 95% CI 0.93–1.00, *p* = 0.047). Additionally, higher circulating WBC counts, particularly neutrophil counts, were associated with a suggestive decrease in hip OA (WBC IVW: OR = 0.94, 95% CI 0.89–0.99, *p* = 0.015; neutrophil IVW: OR = 0.93, 95% CI 0.88–0.99, *p* = 0.017). Conversely, reverse MR analysis found no evidence to support a genetic effect of OA on circulating WBC counts. **Conclusion**: Our findings suggest that elevated neutrophil counts may offer protective effects against OA, underscoring the interplay between the immune functions and OA pathogenesis. *CD4*, *CSF1R*, and *TYROBP* emerge as promising OA biomarkers, meriting further validation in prospective studies.

## 1. Introduction

Osteoarthritis (OA) is a prevalent degenerative disease that affects hundreds of millions globally [1]. OA results in degenerative changes in joint-related tissues and mild systemic inflammation; in severe cases, it can lead to disability, significantly affecting patients’ quality of life and lifespan [2]. Although OA can affect any joint, hip and knee OA are the most commonly observed types in clinical practice [3]. Due to the heterogeneous nature of OA, patients experience varying levels of disease severity, which necessitates long-term management and treatment [4]. Current guidelines recommend a combination of pharmacological and non-pharmacological approaches, with particular emphasis on non-drug strategies such as a healthy diet and weight loss for the treatment of OA across different severity levels [5,6,7]. However, effective therapeutic options that can significantly improve the condition and meet patient expectations remain scarce [5,6]. While studies suggest factors like trauma, obesity, and low-grade inflammation may contribute to the occurrence of OA [8], the specific pathogenesis remains poorly understood. Given the substantial economic burden that the diagnosis and treatment of OA place on society, an urgent need exists to explore OAs’ specific etiology and develop novel diagnostic markers and treatment approaches [9].

Transcriptome-based gene expression profiling has recently become widely used in biomedical and clinical research, particularly for identifying disease biomarkers [10]. For example, in oncology research, transcriptome data from both tumors and physiological samples enable researchers to identify tumor-associated biomarkers. This approach allows researchers to detect differentially expressed genes that may serve as potential biomarkers for diagnosis, prognosis, and treatment response. Numerous studies have utilized this method to identify biomarkers across diverse tumor types [11]. Similarly, OA has been investigated using comparable approaches in prior studies. For instance, a recent study identified three tryptophan metabolism-related biomarkers in OA-*TDO2*, *AOX1*, and *SLC3A2* through transcriptome analysis [12]. In the present study, we integrated recently published transcriptome data on OA with various bioinformatics methods to identify OA-related biomarkers and performed functional enrichment analysis on these biomarkers. Preliminary results indicate a strong correlation between OA-related genes and white blood cells (WBCs), which are a crucial component of the immune system. This supports the recently revised understanding of OA pathogenesis, where the immune system plays a significant role in disease development. Traditionally, OA was considered a degenerative joint disease caused by mechanical wear and tear. However, contemporary research suggests that OA is accompanied by chronic low-grade inflammation, primarily driven by the innate immune system [1]. Joint damage triggers the release of damage-associated molecular patterns, which activate the innate immune response and lead to the infiltration and activation of immune cells such as macrophages [13]. These cells produce proinflammatory cytokines (e.g., IL-1β and TNF-α), which accelerate cartilage degradation [13,14]. Moreover, components of the adaptive immune system, such as T cells and B cells, also contribute to the pathological process of OA, exacerbating the inflammatory response [14,15]. These insights motivated us to further investigate the genetic association between circulating WBCs and OA.

Previous studies have found that the WBCs in OA patients vary and are closely linked to OA diagnosis and treatment. For example, the WBC counts in the synovial fluid of knee of OA patients may indicate inflammation severity [16]. Leukocyte-rich, platelet-rich plasma demonstrates significant anti-inflammatory effects in patients with mild to moderate knee OA [17]. Additional, Manukyan et al. confirms the prominent role of neutrophils in knee OA pathophysiology [18]. Although WBCs in OA originate from the circulatory system, the relationship between circulating WBC counts and OA pathogenesis remains still unclear. Undoubtedly, randomized controlled trials (RCTs) are regarded as the gold standard for assessing causal effects [19], but their implementation can be practically challenging. The mendelian randomization (MR) approach parallels RCTs by assessing the potential causal impact of traits on diseases through the random allocation of genetic variations at conception. In epidemiological research, the MR method can mitigate biases found in observational studies, yielding more reliable results and reducing the influence of confounding factors and reverse causation [20,21]. Recent studies have used MR to explore traits potentially associated with OA risk [3,22,23,24], yet no MR study has investigated the genetic relationship between circulating WBC counts and OA. This study aims to employ bioinformatics analysis in conjunction with a bidirectional, two-sample MR approach to identify genetic markers associated with OA, and to preliminarily investigate the potential genetic relationship between circulating WBC count and OA.

## 2. Materials and Methods

### 2.1. Data Sources

The most recent OA microarray dataset (GSE236924) was obtained from the Gene Expression Omnibus (GEO) database and includes normalized gene expression data of 89 OA patients and 7 controls [25]. Additionally, the GSE114007 cohort was also obtained from the GEO database as a validation cohort, comprising normalized RNA-Seq data of 20 OA and 18 normal samples [26]. The data sources and characteristics of genome-wide association study (GWAS) cohorts used in this study are summarized in Table 1. GWAS summary data for OA at any site, hip, and knee OA were obtained from the study by Boer et al. [27], accessible through The Musculoskeletal Knowledge Portal (https://mskkp.org) (accessed on 1 December 2023). This meta-analysis represents the largest genome-wide study for OA to date, including data from 826,690 individuals. For the current study, summary-level GWAS data from OA at any site (n = 177,517), hip OA (n = 36,445), and knee OA (n = 62,497) were used. To minimize bias, all participants in the selected datasets were restricted to European ancestry. Summary GWAS data of circulating WBC counts were sourced from the latest study by Chen et al. [28], which included 563,946 participants. We used summary-level GWAS data for the total WBC counts and for counts of five subtypes (lymphocytes, monocytes, neutrophils, basophils, and eosinophils), available through IEU Open GWAS (https://gwas.mrcieu.ac.uk/) (accessed on 10 December 2023) under accession numbers ieu-b-29 to ieu-b-34. As all data used in this study were obtained from previously published sources and public databases, no additional ethical approval or informed consent was required.

### 2.2. The Identification of Differentially Expressed Genes (DEGs)

Linear models for microarray data (Limma) is a differential expression analysis method that utilizes generalized linear models. In this study, we used the R package “Limma” (version 3.58.1) [29] to perform differential expression analysis and identify DEGs between OA and control samples in GSE236924. The screening thresholds applied in this study were a *p*-value < 0.05 and |fold change (FC)| > 2.

### 2.3. The Identification of Weighted Gene Co-Expression Network Analysis Hub Genes

In this study, the R package “WCGNA” (version 1.72-5) was used to identify hub genes through weighted gene co-expression network analysis (WGCNA) [30]. First, gene expression profiles were used to calculate the median absolute deviation (MAD) of each gene, and the top 50% of genes with the smallest MAD were removed. Next, the “goodSamplesGenes” function in the “WGCNA” package was applied to remove outlier genes and samples. WGCNA was then used to construct a scale-free co-expression network, with the soft threshold determined based on network scale-free topology and mean connectivity. After selecting the soft threshold, the adjacency matrix was transformed into a topological overlap matrix (TOM), to measure the network connectivity, and the corresponding dissimilarity (1-TOM) was calculated. Average linkage hierarchical clustering was performed based on TOM-derived dissimilarity to generate a gene dendrogram. Modules with a distance threshold below 0.25 were merged. Following previous studies [11], gene modules with correlation coefficients |r| > 0.5 were identified as OA-related WGCNA gene modules, and their genes were designated as WGCNA hub genes.

### 2.4. Enrichment Analysis

Gene ontology (GO) and Kyoto encyclopedia of genes and genomes (KEGG) enrichment analyses were performed using the R package “clusterProfiler” (version 4.8.3) [31]. A *p*-value of <0.05 and a false discovery rate (FDR) value of <0.05 were set as thresholds for statistical significance. The GO enrichment analysis covered three categories: biological processes, molecular functions, and cellular components.

### 2.5. The Identification of Key OA-Related Genes

First, the intersection of differentially expressed genes (DEGs) and WGCNA hub genes was submitted to the STRING database (https://string-db.org/) (accessed on 26 November 2023) [32] to construct the protein–protein interaction (PPI) network using default parameters. The PPI network was visualized using Cytoscape software (version 3.9.0). Additionally, three algorithms (betweenness, closeness, and degree) in Cytoscape were used to identify the top 30 hub genes in the PPI network, with the intersection of the hub genes across these algorithms defined as the key hub gene. Finally, the random forest algorithm was applied to identify the most significant key hub genes, namely key OA-related genes, based on mean decrease accuracy.

### 2.6. Study Design of MR Analysis

**Figure 1:**
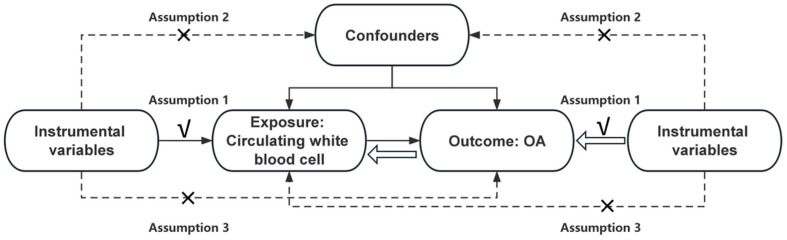
Mendelian randomization study design.

This study adhered to the three key assumptions of MR in two-sample MR analysis: (1) IVs are significantly associated with the exposure; (2) IVs are independent of any confounders affecting the relationship between exposure and outcome; (3) IVs are not directly related to the outcome [33]. A bidirectional two-sample MR analysis was performed to investigate the potential causal relationship between circulating WBC counts and OA. First, a two-sample MR analysis was conducted using circulating WBC counts as the exposure and OA as the outcome. Then, a reverse two-sample MR analysis was performed, treating OA as the exposure and circulating WBC counts as the outcome. Figure 1 provided an overview of the bidirectional MR design and the three key MR assumptions.

### 2.7. Selection of SNPs

In this study, a *p*-value threshold of <5 × 10^−8^ was applied as the genome-wide significance level for single-nucleotide polymorphism (SNP) selection, with circulating WBC counts as exposure and OA as exposure. To mitigate linkage disequilibrium among IVs, a clumping distance of 10,000 kb and R2 threshold of <0.001 were applied in the SNP selection process. Additionally, to prevent weak instrument bias, SNPs with F statistics < 10 were excluded. Harmonization of SNPs between the exposure and outcome variables was performed to ensure alignment with the same alleles. SNPs strongly associated with the outcome (*p* < 5 × 10^−5^) and palindromic SNPs were excluded. Steiger tests were conducted to rule out reverse causality, and SNPs failing the test were excluded. Furthermore, the PhenoScanner database (http://phenoscanner.medschl.cam.ac.uk/) (accessed on 26 November 2023) was queried to identify an association between SNPs and potential confounders, such as obesity; SNPs linked to confounders were excluded. In the forward MR analysis, 169~438 SNPs were included in this study for circulating WBC counts for OA at any site, 168~439 SNPs for hip OA, and 170~438 SNPs for knee OA. For the reverse MR analysis, this study included 16~18 SNPs related to OA at any site, 20~21 SNPs for hip OA, and 14~16 SNPs for knee OA for circulating WBC counts. A summary of all SNPs used is provided in Supplementary Table S1.

### 2.8. Statistical Analysis

All statistical analyses were performed using R software (version 4.3.3). Group differences were analyzed using the Wilcoxon rank-sum test, with a significance threshold set at *p* < 0.05. The R package “pROC” (version 1.18.5) was used to generate receiver operating characteristic (ROC) curves and calculate the area under the curve (AUC). Four MR analysis methods were applied to assess the genetic relationship between circulating WBC counts and OA: inverse variance-weighted (IVW) [34], MR-Egger regression [35], weighted median [36], and weighted mode [37]. Previous research has indicated that the IVW method is more robust than other methods under certain conditions [36]. Consequently, this study focuses on the IVW results, while the other three methods serve as supplementary analyses. Horizontal pleiotropy was evaluated using the MR-Egger intercept, with a *p*-intercept < 0.05 suggesting significant pleiotropy. Cochran’s Q test within the IVW framework was conducted to evaluate heterogeneity among the included SNPs in each analysis, with a *p*-value < 0.05 indicating substantial heterogeneity [38]. A leave-one-out analysis was performed to detect potential SNP outliers. The TwoSampleMR (version 0.5.7) and MendelianRandomization (version 0.8.0) packages were used for all MR analyses. A suggestive genetic association was defined by a *p*-value < 0.05. Given the exploratory nature of this study, adjusted *p*-values were not applied in the MR analysis.

## 3. Results

### 3.1. The Identification of DEGs and WGCNA Hub Genes

**Figure 2:**
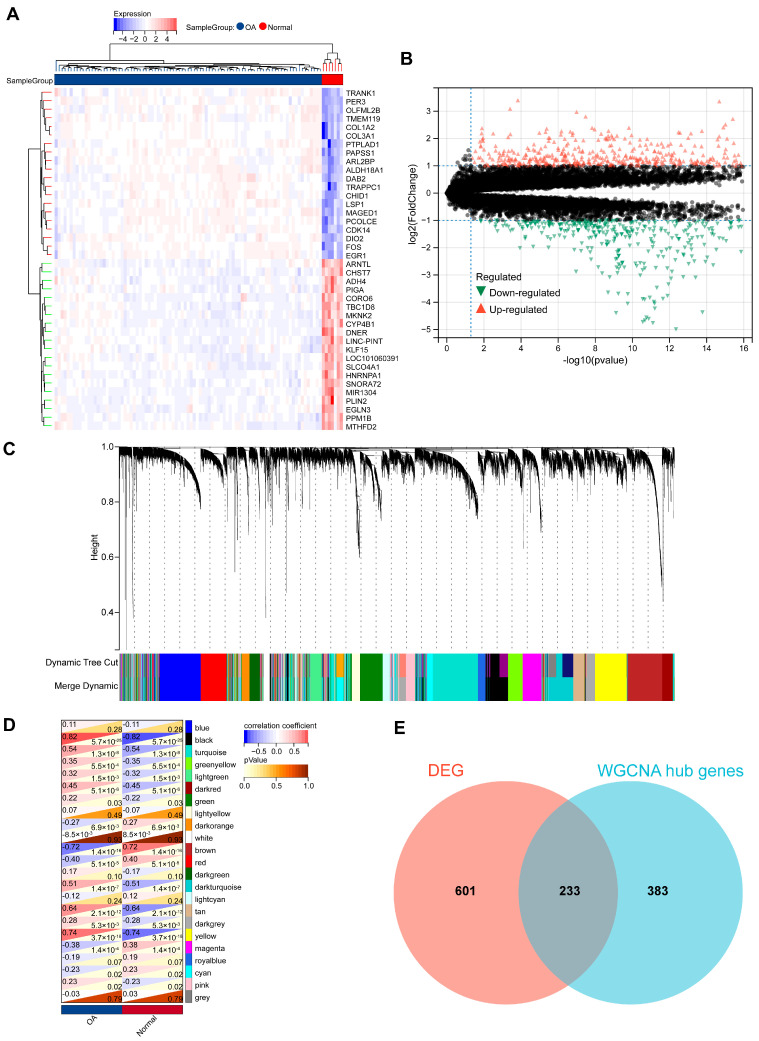
The identification of DEGs and WGCNA hub genes. (**A**) The heatmap displays the expression of the top 20 upregulated (red) and downregulated (blue) genes in OA. (**B**) Volcano plot showing all DEGs between the OA group and the normal group, with significantly upregulated (red) and downregulated (green) genes. (**C**) Gene modules identified by WGCNA. (**D**) Correlation analysis between gene modules and OA: positive (red) and negative (blue) correlations. (**E**) Intersection of 834 DEGs and 616 WGCNA hub genes.

Comparison between the OA group and the normal group in the GSE236924 cohort identified 834 DEGs, with 472 DEGs upregulated and 362 DEGs downregulated in the OA group (Supplementary Table S2). Figure 2A presents a heatmap of the top 20 upregulated and downregulated genes, while Figure 2B displays all DEGs in a volcano plot. Using a soft threshold to seven in the WGCNA analysis, based on scale-free and mean connectivity network maps (Supplementary Figure S1), we identified 22 co-expressed gene modules (Figure 2C). Correlation analysis revealed that the black, turquoise, dark turquoise, tan, and yellow modules were significantly positively correlated with OA, while the brown module was significantly negatively correlated with OA (Figure 2D). Together, these modules included 616 genes, referred to as WGCNA hub genes (Supplementary Table S3). The intersection of 834 DEGs and 616 WGCNA hub genes yielded 233 OA-related genes (Figure 2E, Supplementary Table S4).

### 3.2. Functional Enrichment Analysis of OA-Related Genes

**Figure 3:**
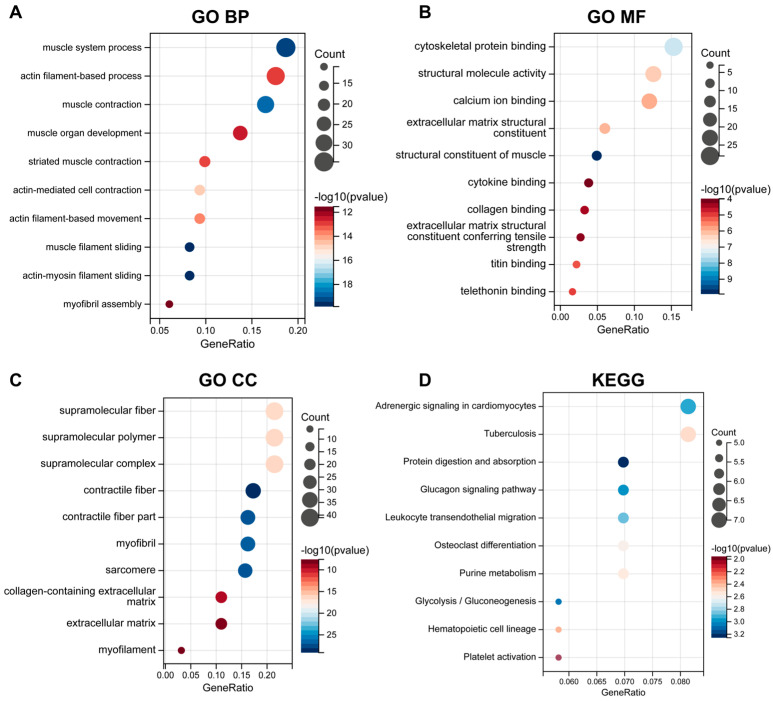
Enrichment analysis of OA-related genes. (**A**–**C**) Bubble plots depicting GO enrichment in biological process terms (**A**), molecular function terms (**B**), and cellular component terms (**C**). (**D**) Bubble plot illustrating KEGG pathway analysis.

Functional enrichment analysis was conducted on the 233 OA-related genes to investigate their potential biological roles. GO biological process terms showed that these genes were predominantly enriched in muscle system-related processes, including “actin filament-based process”, and “muscle filament sliding” (Figure 3A). In GO molecular function terms, the genes were enriched in matrix-related functions, including “cytokine binding”, “extracellular matrix structural constituent”, and “collagen binding” (Figure 3B). GO cellular component terms showed enrichment in the “extracellular matrix”, along with supramolecule-related cellular components (Figure 3C). Notably, OA-related genes were also enriched in WBC-related KEGG pathways, including “leukocyte transendothelial migration” and “hematopoietic cell lineage” (Figure 3D).

### 3.3. The Identification of Key OA-Related Genes

**Figure 4:**
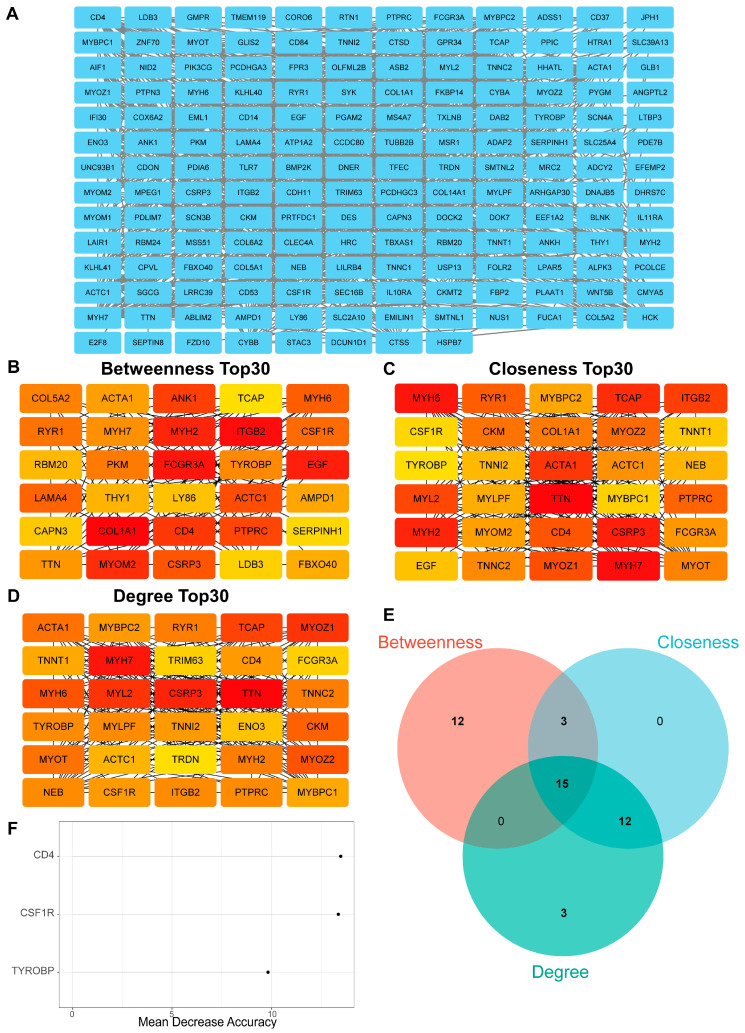
Construction of the PPI network and selection of hub genes. (**A**) The PPI network of 156 OA-related genes, constructed using STRING, excluding 77 DEGs due to a lack of interaction. (**B**–**D**) Betweenness (**B**), closeness (**C**), and degree (**D**) algorithms were employed to select the top 30 hub genes in Cytoscape, with darker colors indicating greater significance. (**E**) Intersection of the top 30 hub genes from betweenness, closeness, and degree algorithms. (**F**) Mean decrease accuracy, based on random forest algorithm, was used to rank the predictive importance of OA-related genes and identify the three most critical key OA-related genes.

Initially, a preliminary PPI network was constructed from the 233 OA-related genes to identify potential hub genes (Supplementary Figure S2). Seventy-seven genes without connections to other genes were excluded, resulting in a PPI network constructed with 156 genes (Figure 4A). Three Cytoscape plug-in algorithms were used to further refine and identify the top 30 hub genes within the PPI network. Figure 4B–D displays the top 30 hub genes in the three algorithms. A Venn plot illustrates the overlap among the top 30 hub genes identified by the three algorithms (Figure 4E), revealing 15 overlapping genes as the final hub genes (Supplementary Table S5). Using the random forest algorithm, we assessed the importance of 15 hub genes, ultimately identifying three key OA-related genes—*CD4*, *CSF1R*, and *TYROBP*—as potential diagnostic biomarkers for OA (Figure 4F).

### 3.4. The Diagnostic Value of Key OA-Related Genes

**Figure 5:**
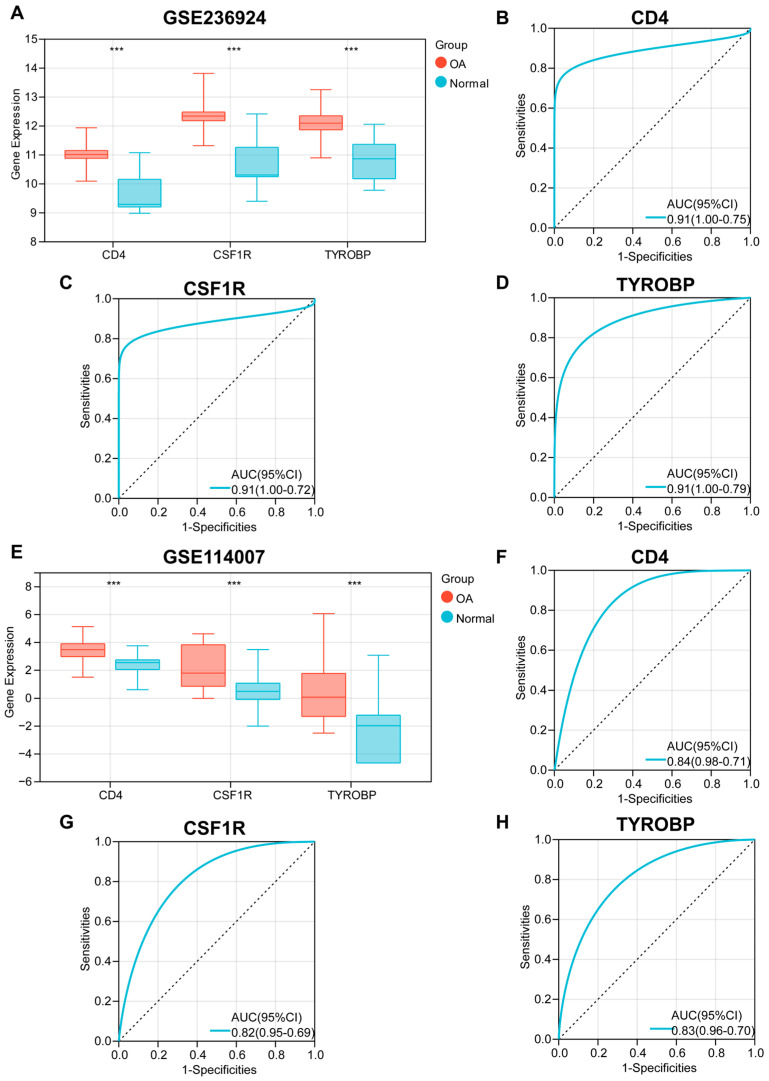
Evaluation of the diagnostic value of three key OA-related genes. (**A**) Expression differences of *CD4*, *CSF1R*, and *TYROBP* between OA and normal tissues in the GSE236924 cohort. (**B**–**D**) The ROC curves used to evaluate the diagnostic efficacy of *CD4* (**B**), *CSF1R* (**C**), and *TYROBP* (**D**) in identifying OA in the GSE236924 cohort. AUC and 95% CI presented in each panel. (**E**) Expression differences of *CD4*, *CSF1R*, and *TYROBP* between OA and normal tissues in the GSE114007 cohort. (**F**–**H**) The ROC curves used to evaluate the diagnostic efficacy of *CD4* (**F**), *CSF1R* (**G**), and *TYROBP* (**H**) in identifying OA in the GSE114007 cohort. AUC and 95% CI presented in each panel. ***, *p* < 0.001.

In the GSE236924 cohort, OA samples exhibited significantly upregulated expression of *CD4*, *CSF1R*, and *TYROBP* compared to normal tissues (Figure 5A). ROC curve analyses demonstrated satisfactory diagnostic efficacy for each of the three genes, *CD4*, *CSF1R*, and *TYROBP*. Figure 5B–D illustrates the AUC and 95% confidence intervals (CI) for each gene: *CD4* (AUC: 0.91, 95%CI: 0.75–1.00), *CSF1R* (AUC: 0.91, 95%CI: 0.72–1.00), and *TYROBP* (AUC: 0.91, 95%CI: 0.79–1.00). To further validate the diagnostic utility of the three genes, analyses were conducted on an independent validation dataset. In the GSE114007 cohort, the expressions of all three genes were also significantly upregulated in OA samples (Figure 5E). ROC curves confirmed robust diagnostic performance for the three genes in the GSE114007 cohort as well (Figure 5F–H).

### 3.5. Genetic Effects of Circulating White Blood Cells on OA

**Figure 6:**
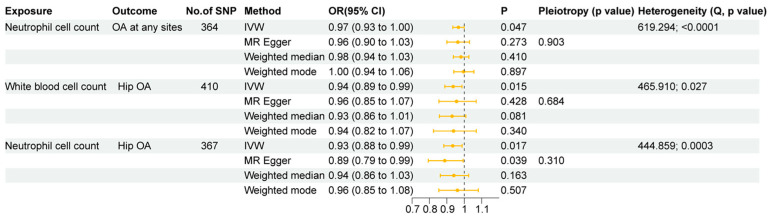
Causal effects of circulating white blood cells on OA: positive mendelian randomization Results.

**Figure 7:**
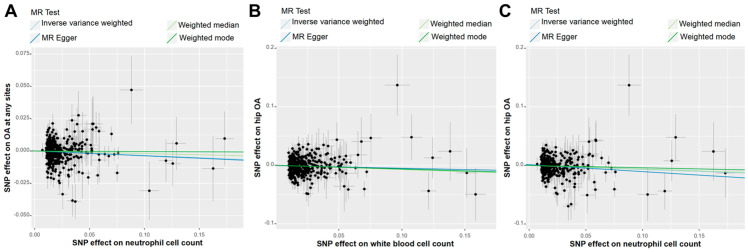
The scatter plots of causal effects. Scatter plots depicting the causal effects of neutrophil cell count on OA at any site (**A**), total white blood cell count on hip OA (**B**), and neutrophil cell count on knee OA (**C**).

Using forward two-sample MR analysis, we examined the genetic effects of circulating WBC counts on OA, specifically for OA at any site, hip OA, and knee OA. The results are summarized in Supplementary Tables S6–S8. Figure 6 shows that IVW analysis indicated a negative genetic effect of neutrophil cell count on OA at any site (OR: 0.97, 95%CI: 0.93~1.00, *p* = 0.047), with support from additional methods. Both neutrophil cell count (OR: 0.93, 95%CI: 0.88~0.99, *p* = 0.017) and total WBC count (OR: 0.94, 95%CI: 0.89~0.99, *p* = 0.015) exhibited negative genetic effects on hip OA, with additional methods indicating the same association direction as the IVW analysis. Figure 7 provides scatter plots illustrating the aforementioned genetic associations. However, no evidence was found to suggest a genetic relationship between circulating WBC counts and knee OA (Supplementary Table S8).

Subsequently, the presence of horizontal pleiotropy in the MR analysis was assessed, with the MR-Egger intercept showing no evidence of horizontal pleiotropy in genetic effects (Figure 6, all *p* > 0.05). The IVW heterogeneity test indicated heterogeneity in three of the genetic associations above. In addition, the leave-one-out analysis confirmed that no outlier SNPs were present (Supplementary Tables S9–S11).

### 3.6. Genetic Effects of OA on Circulating White Blood Cells

To evaluate potential reverse genetic effects, we conducted two-sample MR analysis using OA at any site, hip OA, and knee OA as exposures, with circulating WBC counts as the outcome. While the weighted median, weighted mode, and MR-Egger methods detected genetic effects of OA on specific circulating WBC counts—such as a positive genetic effect of OA at any site on total WBC count identified by the weighted median method (Supplementary Table S12)—the IVW method did not provide evidence supporting a genetic effect of OA on circulating WBC counts (Supplementary Tables S12–S14). Since this study relies on the IVW method as a primary reference, these findings do not support the genetic effect of OA on circulating WBC counts.

## 4. Discussion

OA, the most commonly diagnosed joint disorder, primarily affects cartilage and adjacent tissues [39]. In advanced stages, OA is characterized by the breakdown and eventual loss of joint cartilage, along with alterations in subchondral bone, including osteophyte development, decreased strength in surrounding muscles, and increased ligament laxity. OA commonly affects joints in the hands, hips, knees, feet, and spinal regions, but it can also affect other joints [39]. The high prevalence of occurrence and debilitating impact of OA have motivated researchers to develop new biomarkers and investigate potential risk factors. In this context, we employed bioinformatics analysis to identify OA biomarkers and used MR to investigate the potential causal relationship between WBC counts and OA.

Reliable biomarkers are essential in modern medicine. The application of bioinformatics has greatly advanced the exploration of underlying mechanisms and the discovery of biomarkers. Bioinformatic methods facilitate the precise identification of disease-related biomarkers, enhancing our understanding of disease onset and progression revealing potential pathogenic mechanisms. In this study, we used the “Limma” package and WGCNA method, combined with differential expression and phenotype correlation analyses, to comprehensively identify 233 OA-related genes. Subsequent enrichment analyses indicated that OA-related genes are strongly associated with biological processes involving the muscular system, extracellular matrix, and WBCs. This finding aligns with previous research, which suggests that, although OA is traditionally viewed as a wear and tear disease, recent insights highlight immune system involvement, particularly WBCs, in OA pathogenesis [15,40,41]. To investigate interactions among OA-related genes, we constructed a PPI network using the STRING database. We then applied three distinct algorithms to identify hub genes within the network. Machine learning, as a flexible prediction approach, often achieves greater accuracy than compared to traditional regression methods [42]. Using the random forest algorithm, a type of machine learning, we identified three potential OA-related biomarkers from the hub genes: *CD4*, *CSF1R*, and *TYROBP*. Analysis of two independent cohorts confirmed the diagnostic value of *CD4*, *CSF1R*, and *TYROBP* for OA and also established their reliability as OA biomarkers. It is noteworthy that the biomarkers identified in our study demonstrate superior diagnostic performance compared to the currently recognized OA biomarkers, including *COMP*, *MMP3*, *COL2A1*, and *CXCR2* (Supplementary Figure S3) [43,44]. This suggests that these biomarkers are promising diagnostic candidates and may also serve as potential targets for further development of personalized therapeutic strategies for OA.

*CD4* is a glycoprotein mainly expressed on the surface of immune cells, especially T-helper cells. As a key immune marker, *CD4* is essential in regulating immune responses and maintaining immune homeostasis [45]. Studies have shown that in OA, CD4+ T cell infiltration predominates in synovial tissue, with significantly higher CD4+ T cell counts in the subsynovial layer of OA patients compared to normal controls. Additionally, OA patients with knee involvement show a higher CD4+ T cell count in the medial synovium compared to the lateral synovium [46]. The joint’s inflammatory environment activates CD4+ T cells, potentially worsening cartilage degeneration and joint pain [47]. Proinflammatory cytokines from activated CD4+ T cells can affect chondrocyte and synovial cell function, promoting a catabolic state that accelerates articular cartilage degeneration [13,48]. This immune-mediated pathway underscores the potential of targeting CD4+ T cells in therapies to mitigate OA.

*CSF1R*, a cell surface receptor central to the proliferation, differentiation, and survival of monocytes/macrophages, is crucial in immune and inflammatory responses [49]. Studies indicate that macrophages constitute around 65% of immune cells infiltrating the synovial tissue of OA patients [50]. Recent findings suggest *CSF1R* as a key molecular target in OA pathogenesis, with significantly elevated expression in OA [51,52], highlighting its potential as a therapeutic target. Moreover, intra-articular blockade of *CSF1R* effectively alleviates knee joint edema and pain in OA patients [53]. These studies provide compelling evidence supporting the role of *CSF1R* in OA diagnosis and treatment.

*TYROBP*, also known as *DAP12* or *KARAP*, is a transmembrane adaptor protein originally identified as a receptor activation subunit in NK cells. Recent studies have shown *TYROBP* expression in various WBCs, including peripheral blood monocytes, macrophages, and dendritic cells, all key components of innate immunity [54]. Emerging theories on OA pathogenesis propose that OA results from the interaction between mechanical injury and chronic inflammation [55], with innate immune system activation playing a key role in initiating and sustaining this low-grade chronic inflammation [56,57]. These findings implicate a potential role for *TYROBP* in the development of OA. Indeed, Yan et al. have also identified *TYROBP* as a diagnostic marker for OA [58]. Collectively, multiple studies confirm the feasibility of *CD4*, *CSF1R*, and *TYROBP* as OA biomarkers.

The enrichment analysis results indicated a potential association between OA-related genes and WBC function. Key OA-related genes identified in this study are also well-established biomarkers for WBCs, including *CD4*, a T cell biomarker, and *CSF1R*, a biomarker for monocytes. Previous studies have further confirmed that *TYROBP* is associated with WBC function, specifically in NK cells [59,60]. These associations prompted further investigation into the potential genetic relationship between circulating WBCs and OA. This study uses MR analysis and extensive GWAS data to explore the genetic relationship between circulating WBC counts and OA in European populations. As the most abundant WBC type, neutrophils are traditionally recognized for their role in rapidly initiating immune responses to inflammation and injury. Within the context of OA, neutrophil function has been increasingly studied, revealing a more complex role than previously understood [61,62,63]. Our genetic analysis provided evidence of a genetic association between elevated neutrophil cell counts and a reduced risk of OA at any site; similarly, increased circulating WBC and neutrophil cell counts showed a suggestive association with a decreased risk of hip OA.

The bidirectional design of our MR analysis is crucial for unraveling the complex interactions between immune system components and OA. The forward MR analysis indicated a negative genetic relationship between neutrophil and total WBC counts with OA, particularly in the hip, whereas the reverse MR analysis found no evidence of a genetic effect of OA on circulating WBC counts. This suggests that changes in WBC counts, especially neutrophils, may be more likely a contributing factor to OA rather than a consequence. Interestingly, no genetic relationship was found between circulating WBC counts and knee OA. This finding may indicate distinct pathophysiological mechanisms underlying knee OA as opposed to hip OA, highlighting the need for more detailed studies on leukocyte subtypes, particularly neutrophil subtypes, and their potential role. Indeed, the structural and biomechanical differences between the hip and knee joints significantly influence their susceptibility to OA. The hip joint, a ball-and-socket joint, allows a wide range of motion and effectively distributes weight during activities such as walking and running. In contrast, the knee joint, as a hinge joint, is subjected to complex loading patterns, including shear forces during stair climbing and squatting, making it more vulnerable to mechanical stress and uneven cartilage wear [1]. Additionally, the hip benefits from strong supporting muscles and ligaments that help maintain stability, while the knee relies heavily on the quadriceps and hamstrings; weakness in these muscles can disrupt knee biomechanics and contribute to OA [1]. Current research suggests that while the relationship between hip joint load and disease progression remains unclear, higher knee joint loads are associated with more advanced structural disease progression [64,65,66]. Furthermore, the inflammatory processes in hip and knee OA may differ, with certain inflammatory markers being more pronounced in hip OA, potentially influencing disease progression [1,66]. In summary, the occurrence of hip OA may be more strongly associated to the WBC count, while mechanical factors in knee OA may play a more important role in the pathogenesis of the disease.

The role of immune cells in the onset and progression of OA remains incompletely understood. While certain neutrophil subtypes within the joint cavity are commonly regarded as pro-inflammatory, the extent of their influence on OA remains only partially defined. For example, a study examining synovial fluid in OA found that the WBC counts in it were higher in patients with moderate to large effusion-synovitis than in those with small or no effusion-synovitis. This finding suggests a relationship between synovial inflammation and OA severity, as indicated by the extent of effusion-synovitis. However, a study also reported that the sensitivity and specificity of synovial fluid WBC counts in identifying effusion-synovitis on MRI were limited [67]. Peripheral blood leukocytes, especially neutrophils, play a central role in immune regulation. For instance, neutrophils and macrophages work in close collaboration at sites of injury. In the inflammation phase, neutrophils enhance macrophage functions that target the elimination of the initial stimulus. During the resolution phase, apoptotic neutrophils emit key signals that reprogram macrophages to promote tissue healing. Moreover, neutrophil-derived microparticles, which trigger anti-inflammatory responses, represent a novel approach to mitigating inflammation. This underscores the multifaceted role of neutrophils in both inciting and resolving inflammatory processes [68]. Another study further highlights the beneficial aspects of neutrophils in inflammation. This research demonstrates that neutrophils facilitate the clearance of cellular debris and apoptotic cells—processes essential for controlling persistent inflammation and promoting tissue repair [69]. This function is particularly important in the context of chronic diseases prevalent in older populations, where ineffective debris clearance can worsen inflammatory conditions.

Among various treatment strategies for OA, platelet-rich plasma (PRP) therapy is emerging as a notable alternative, primarily due to its regenerative capabilities and favorable safety profile. However, a lack of consensus persists in clinical and research communities regarding the optimal PRP formulation for OA treatment, especially concerning the concentration of leukocytes. A recent study highlighted that leukocyte-rich PRP (LR-PRP) significantly enhances the expression of anti-inflammatory agents such as IL-1Ra, IL-4, and IL-8, suggesting that LR-PRP may be more effective in managing chronic, low-grade inflammation in knee OA [17]. This is further supported by Jayaram and colleagues, who demonstrated in animal models that the impact of PRP therapy on OA progression and associated pain may depend on the presence of leukocytes, with LR-PRP exhibiting greater effectiveness in alleviating thermal hyperalgesia post-surgery [70]. Thus, our study carries significant clinical implications, indicating that leukocytes, while traditionally associated with the pathogenesis of OA, may also possess protective roles in the disease. From a genetic perspective, although the specific mechanisms remain unclear, there seems to be a negative genetic relationship between elevated peripheral circulating WBC counts, particularly high neutrophil counts, and decreased incidence of hip OA. Additionally, our study is anticipated to provide valuable insights to the clinical application of PRP therapy in OA management.

However, our study has several limitations. The efficacy of *CD4*, *CSF1R*, and *TYROBP* as biomarkers for OA requires validation in prospective cohorts. The genetic instruments used in the MR analysis serve as proxies for lifetime variations in WBC counts. Consequently, the MR analysis does not permit us to infer how significant fluctuations in WBC counts over short periods may affect the development of OA. Moreover, previous studies have indicated that the WBC count in human blood is influenced by circadian rhythms and gradually increases throughout the day [71]. For instance, neutrophils peak later in the afternoon, while T cells, B cells, and monocytes reach their peak levels around midnight. The WBC count is also influenced by other factors, such as physical exercise [71]. Since the time of blood sample collection by participants was uncertain, the WBC count may have been affected by temporal fluctuations, thereby impacting the interpretation of the results in this study. Further, due to limitations in public databases, we could not determine the relationship between additional subtypes of peripheral WBC counts, particularly neutrophils, and the incidence of OA. We acknowledge that future updates to data may yield different conclusions, and we are committed to remaining vigilant and refining our findings as new data become available. Furthermore, our research exclusively focuses on European populations, which may limit its global applicability. We recognize the critical importance of multi-ethnic studies in providing a broader and more inclusive understanding of genetic contributions to OA. Incorporating diverse populations in future research could uncover population-specific genetic factors and improve the generalizability of our findings.

## 5. Conclusions

In conclusion, our research findings demonstrate a significant association between OA-related genes and WBCs, identifying *CD4*, *CSF1R*, and *TYROBP* as promising biomarkers for OA. Notably, we found that an increase in neutrophils may confer a protective genetic effect against OA, with higher levels of circulating WBCs—particularly elevated neutrophil counts—correlating with a reduced risk of hip OA. These insights enhance our understanding of the underlying mechanism of OA and have the potential to inform future therapeutic strategies. However, these findings necessitate further validation through additional studies, including functional experiments and joint-specific analyses, to elucidate the role of the immune system in the pathogenesis of OA comprehensively.

## Figures and Tables

**Table 1:** Overview of the exposures and outcomes.

Trait	Definition	Population	Cases	Controls	Source
OA at any site	Self-reported and hospital-diagnosed OA at any site	Europeans	177,517	649,173	PMID: 34450027
Hip OA	Hospital-diagnosed hip OA	Europeans	36,445	316,943	PMID: 34450027
Knee OA	Hospital-diagnosed knee OA	Europeans	62,497	333,557	PMID: 34450027
White blood cell	/	Europeans	563,946	/	PMID: 32888493

## Data Availability

The OA datasets GSE236924 and GSE114007 were obtained from the GEO database (https://www.ncbi.nlm.nih.gov/gds) (accessed on 10 December 2023). The GWAS summary data of OA can be downloaded download from The Musculoskeletal Knowledge Portal (https://mskkp.org) (accessed on 1 December 2023). The GWAS summary data of circulating white blood cell can be downloaded from IEU Open GWAS (https://gwas.mrcieu.ac.uk/) (accessed on 10 December 2023). Other relevant data are available from the authors upon reasonable request.

## Supplementary Materials

The following supporting information can be downloaded at: https://www.mdpi.com/article/10.3390/biomedicines13010090/s1, Figure S1: Analysis of network topology for various soft-thresholding powers. A. Scale-free network map. B. Mean connectivity network map; Figure S2: The PPI network of 233 OA-related genes; Figure S3: Diagnostic value of known OA biomarkers; Table S1: All SNPs used in this study; Table S2: DEGs between OA and normal tissue; Table S3: OA-associated module genes in WCGNA analysis; Table S4: The intersection of DEGs and OA-associated module genes; Table S5: The intersection of three algorithms; Table S6: MR results of causal effects of white blood cells on OA at any site; Table S7: MR results of causal effects of white blood cells on hip OA; Table S8: MR results of causal effects of white blood cells on knee OA; Table S9: Leave-one-out analysis results of the causal effect of neutrophil cell count on OA at any site; Table S10: Leave-one-out analysis results of the causal effect of white blood cell count on hip OA; Table S11: Leave-one-out analysis results of the causal effect of neutrophil cell count on hip OA; Table S12: MR results of causal effects of OA at any sites on white blood cells; Table S13: MR results of causal effects of hip OA on white blood cell; Table S14: MR results of causal effects of knee OA on white blood cells.
