# Supplementary material for: Genetic Biomarkers and Circulating White Blood Cells in Osteoarthritis: A Bioinformatics and Mendelian Randomization Analysis
Source: Biomedicines. 2025 Jan 2;13(1):90. doi: 10.3390/biomedicines13010090 (PMC11760900; doi:10.3390/biomedicines13010090)
Supplement: Supplementary file 1 [file biomedicines-13-00090-s001.zip › Supplementary Figures.pdf]

Supplementary Figures

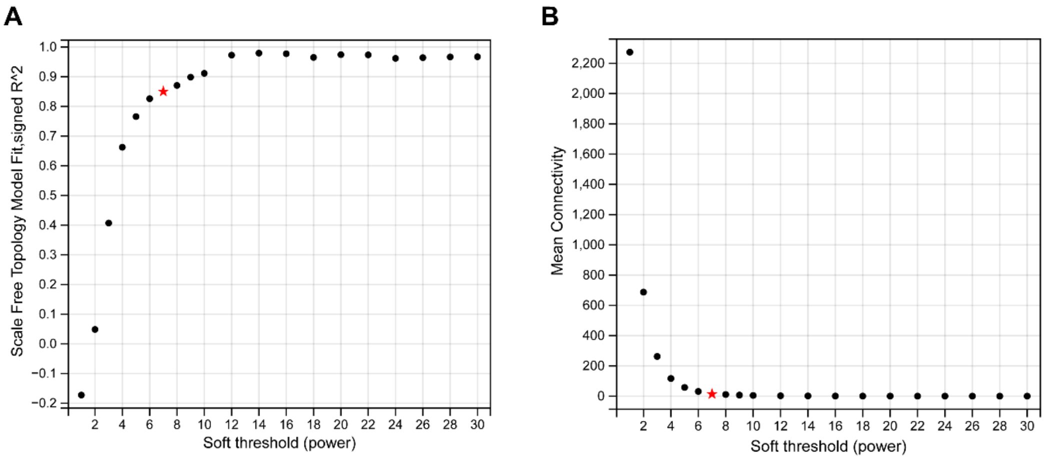

Figure S1. Analysis of network topology for various soft-thresholding powers. A Scale-free network map. **B** Mean connectivity network map.

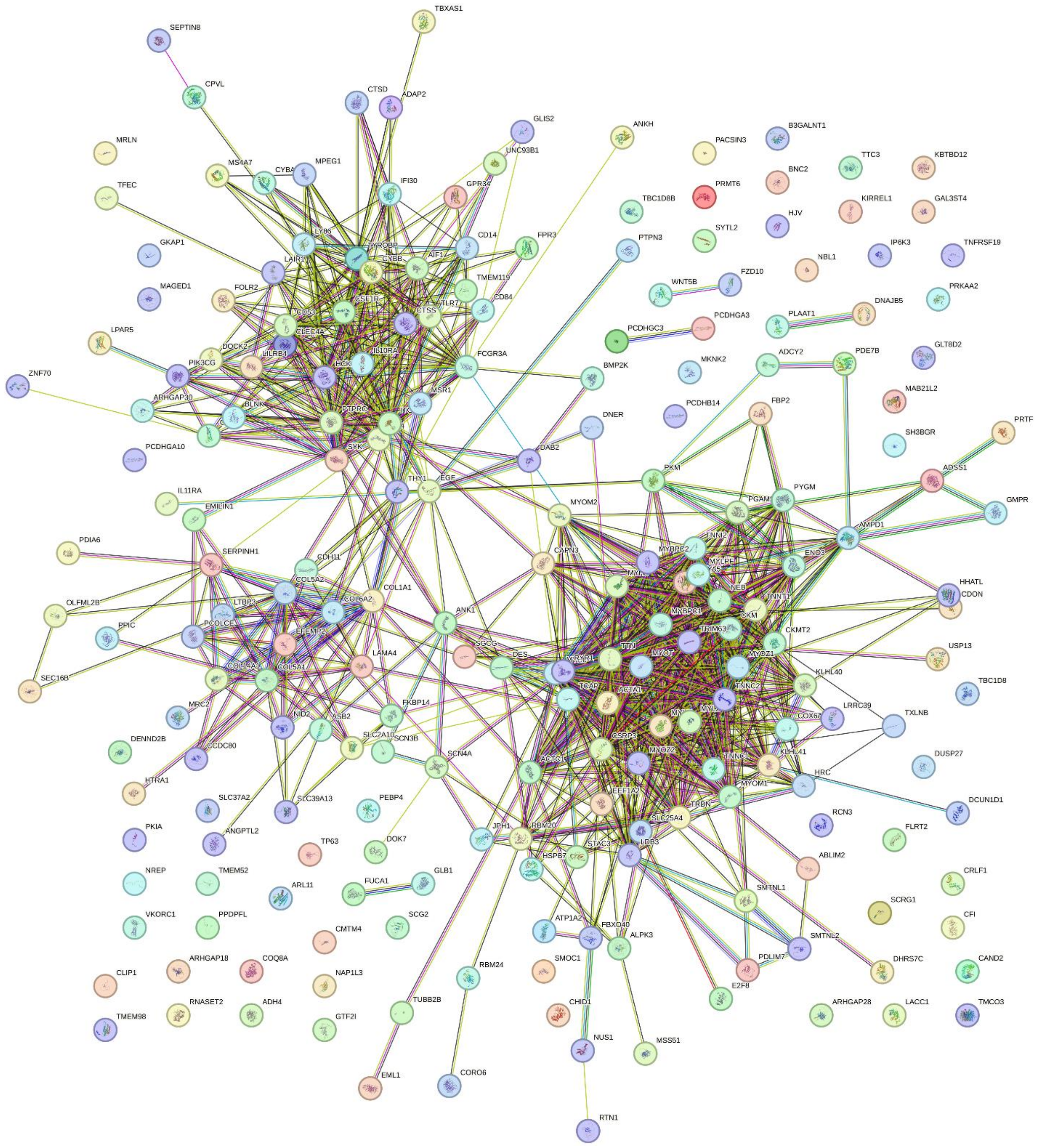

Figure S2. The PPI network of 233 OA-related genes.

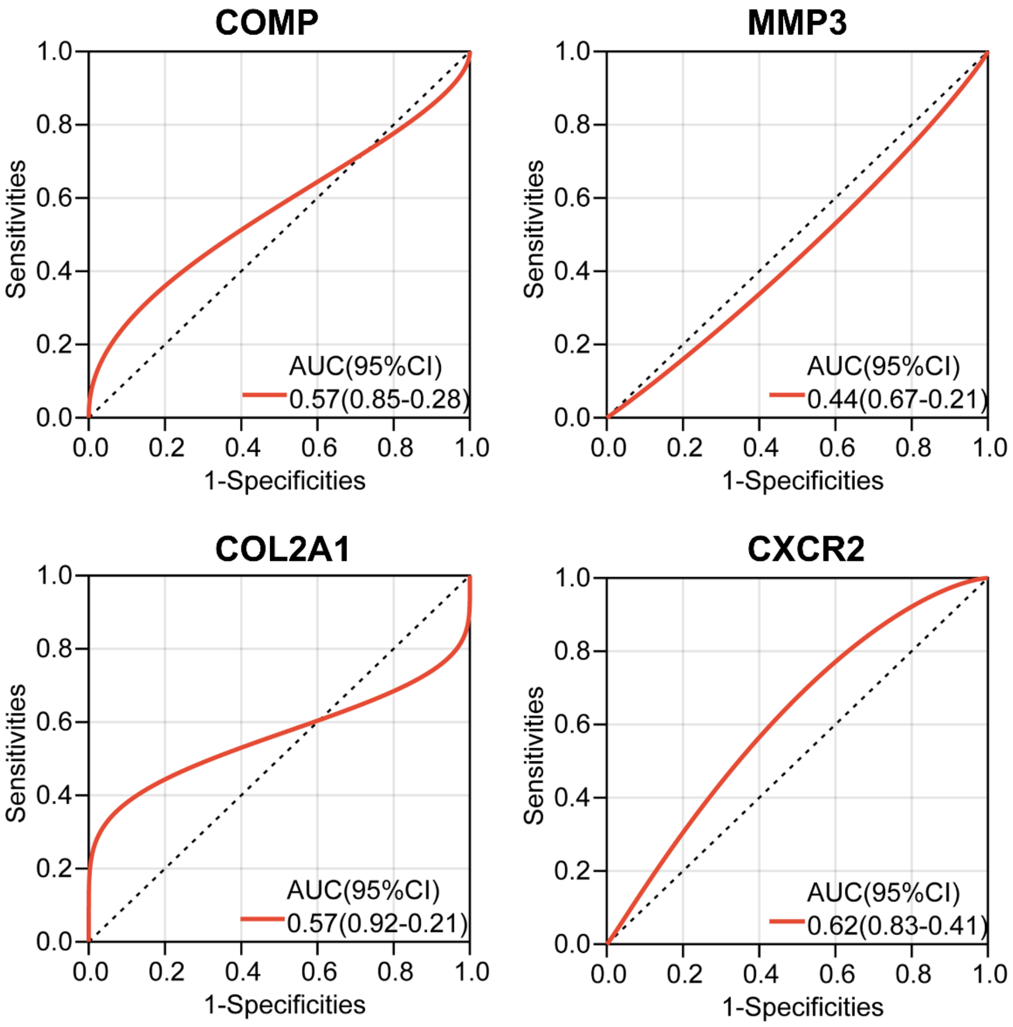

Figure S3. Diagnostic value of known OA biomarkers.
